# Supplementary material for: Multifunctional Hydroxyapatite/Silver Nanoparticles/Cotton Gauze for Antimicrobial and Biomedical Applications
Source: Nanomaterials (Basel). 2021 Feb 8;11(2):429. doi: 10.3390/nano11020429 (PMC7915402; doi:10.3390/nano11020429)
Supplement: Supplementary file 1 [file nanomaterials-11-00429-s001.pdf]

nanomaterials

MDPI

Supplementary Materials

Multifunctional Hydroxyapatite/Silver Nanoparticles/Cotton Gauze for Antimicrobial and Biomedical Applications

Mohamed M. Said [1], Mohamed Rehan [2], Said M. El-Sheikh [3], Magdy K. Zahran [1], Mohamed S. Abdel-Aziz [4], Mikhael Bechelany [5] and Ahmed Barhoum [1,6,*]

1 Chemistry Department, Faculty of Science, Helwan University, 11795 Helwan, Cairo, Egypt; za3balawyscience@gmail.com (M.M.S.); zahranmk@science.helwan.edu.eg (M.K.Z.)
2 Department of Pretreatment and Finishing of Cellulosic based Textiles. Textile Industries Research Division, National Research Centre, 33 Bohoth Street, Dokki, P.O. Box 12622, Giza 12522, Egypt; rehan_nrc@yahoo.com
3 Nanomaterials and Nanotechnology Department, Advanced Materials Division, Central Metallurgical R&D Institute (CMRDI), P.O. Box 87 Helwan, Cairo 11421, Egypt; saidelsheikh@cmrdi.sci.eg
4 Microbial Chemistry Department, Genetic Engineering and Biotechnology Division, National Research Centre, 33 Bohoth Street, Dokki, P.O. Box 12622, Giza 12522, Egypt; mohabomerna@yahoo.ca
5 Institut Européen des Membranes, IEM UMR 5635, Univ Montpellier, CNRS, ENSCM, Montpellier, France; mikhael.bechelany@univ-montp2.fr
6 School of Chemical Sciences, Dublin City University, Dublin 9, Ireland
* Correspondence: ahmed.barhoum@science.helwan.edu.eg or ahmed.barhoum@duc.ie

Figure S1 shows the IR spectrum of chitosan. The strong brand in the region 3291–3361 cm^{-1} corresponds to N–H and O–H stretching, as well as the intramolecular hydrogen bonds. The absorption bands at around 2921 and 2877 cm^{-1} are attributed to C–H symmetric and asymmetric stretching. The presence of residual N-acetyl groups was confirmed by the bands at around 1645 cm^{-1} (-C=O stretching of amide I) and 1325 cm^{-1} (C–N stretching of amide III), respectively. The small band at 1550 cm^{-1} corresponds to the N–H bending of amide II. A peak at 1589 cm^{-1} corresponds to the N–H bending of the primary amine. The –CH_2 bending and –CH_3 symmetrical deformations were confirmed by the presence of bands at around 1423 and 1375 cm^{-1}, respectively. The absorption band at 1153 cm^{-1} can be attributed to asymmetric stretching of the C–O–C bridge. The bands at 1066 and 1028 cm^{-1} correspond to C–O stretching.

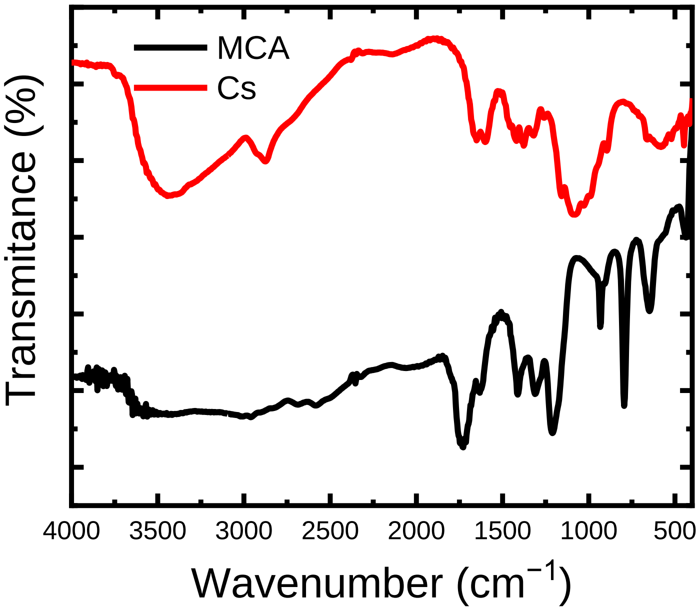

Figure S1. FTIR spectra of pure monochoroacetic acid (MCA) and cationic chitosan (Cs).

Figure S2 shows the Raman spectra of the cotton-Ag and cotton-H5-Ag. The obtained peaks are related to the cellulose chain of the cotton gauze. Cotton gauze samples (cotton-Ag and cotton-H5-Ag) showed strong, well-resolved peaks corresponding to ν of the C–C ring asymmetric stretching and C–O–C for glycoside link asymmetric and symmetric stretching at 1116,1331 and 1088 cm^{-1}, respectively. The other peaks obtained at 1478, 379 and 1292 cm^{-1} are attributed to different types of -CH_2 group vibrations, while peaks at 1337 and 995 cm^{-1} are assigned to C–OH groups (Figure S2a). The peak at 2897 cm^{-1} is assigned to CH and –CH_2 stretching in cellulose. While peak obtained at 2736 cm^{-1} is attributed to the methine group in cotton (Figure S2b). The spectrum of cotton-Ag and cotton-H5-Ag were similar. A new peak that appeared at 540 cm^{-1} is assigned to HAp (Figure S2a).

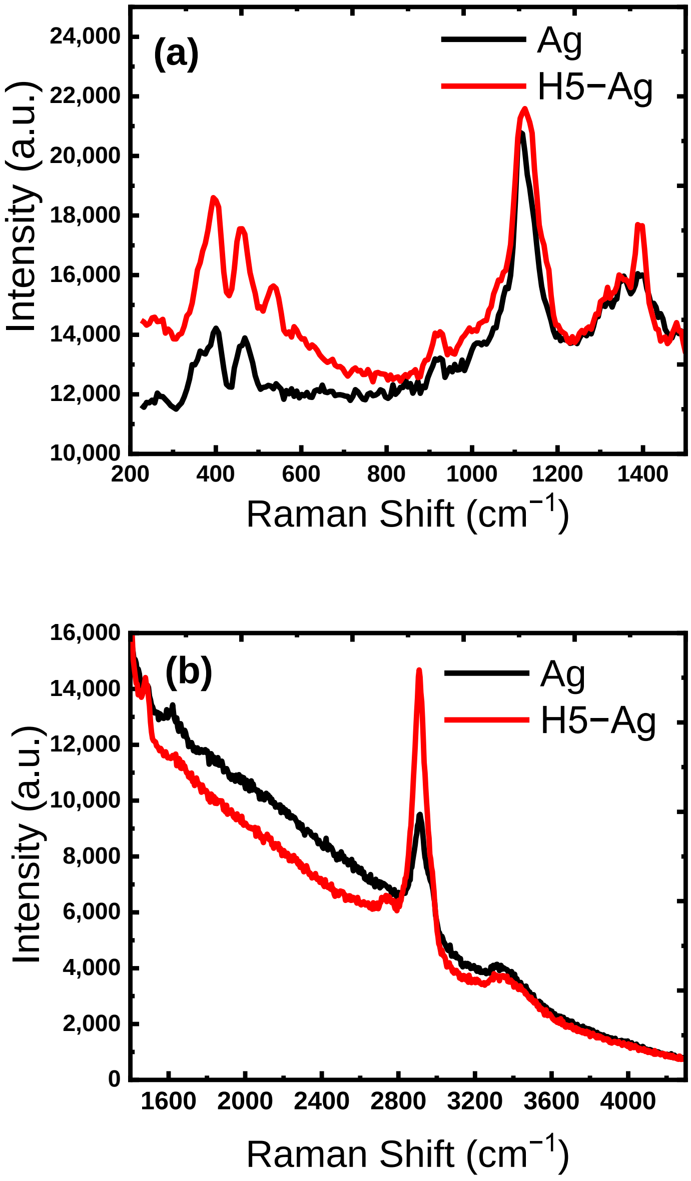

Figure S2. Raman Spectra of the cotton gauze fabrics coated with Ag NPs and HAp-Ag NPs after washing and drying: (**a**) spectrum from 200 to 1500 cm^{-1}; and (**b**) spectrum from 1500 to 4200 cm^{-1}.

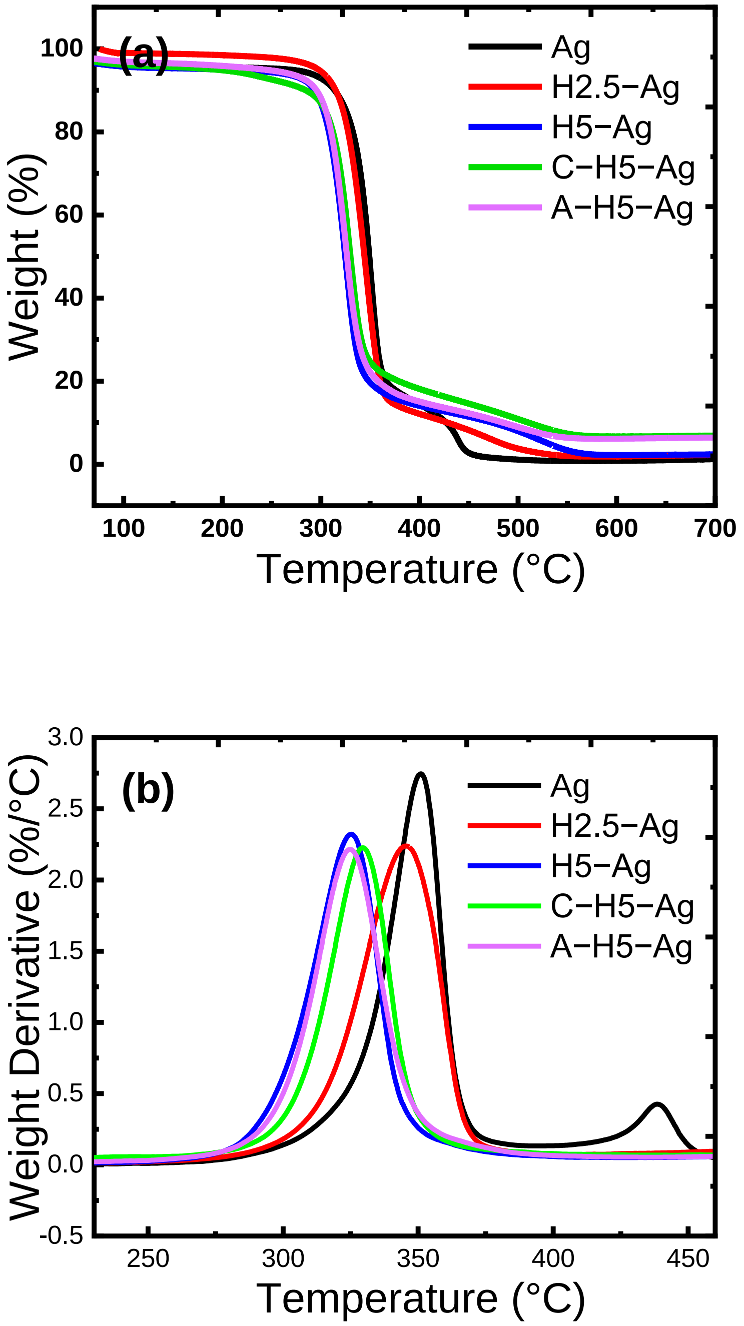

Figure S3. TGA thermal analysis of cotton fiber samples coated with Ag and HAp–Ag NPs: (**a**) TGA weight loss; and (**b**) Weight derivatives. Spectroscopic characteristics of the unmodified (blank) and modified cotton gauze.

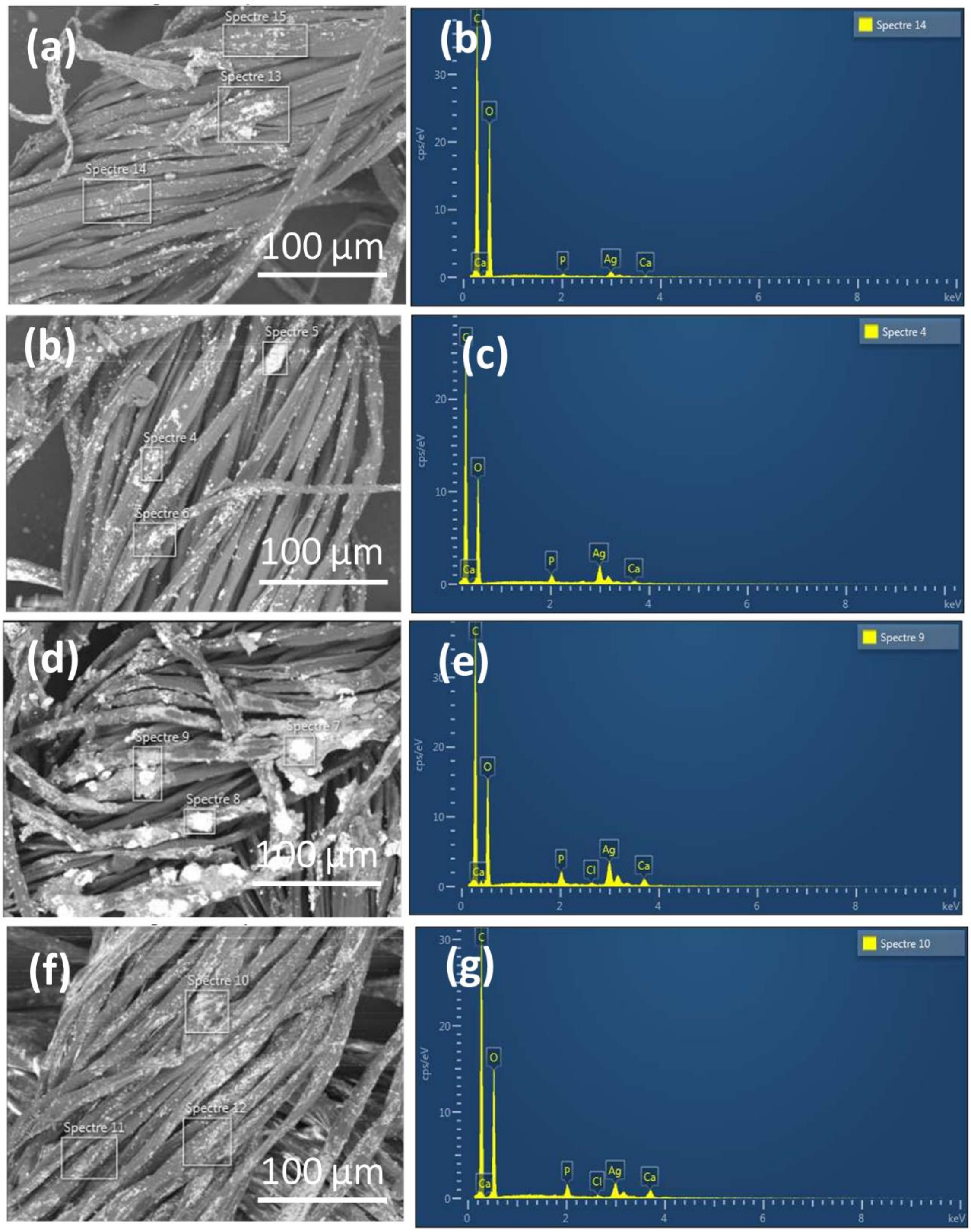

Figure S4. SEM-EDX analysis and their corresponding SEM image of the cotton gauze coated with HAp-Ag NPs: (**a**,**b**) cotton gauze coated with 2.5% HAps and 500 ppm Ag NPs; (**c**,**d**) cotton gauze coated with 5% HAps and 500 ppm Ag NPs; (**e**,**f**) cationic modified cotton gauze coated with 5% HAps and 500 ppm Ag NPs; (**g**,**h**) anionic modified cotton gauze coated with 5% HAps and 500 ppm Ag NPs.

Table S1. Antimicrobial activity, air permeability, water absorption, and tensile strength of the prepared cotton gauze samples.

Sample	Antimicrobial Activity (Inhibition Zone, nm)				Air Permeability	Water Absorption	Tensile Strength
	Gram (+) Bacteria	Gram (−) Bacteria	Fungi	Fungi	cm^3 /cm^2 /sec	% (30 min)	(N/cm^2)
	S. aureus	*E. coli*	*C. albicans*	*A. niger*			
Blank	0	0	0	0	255.3 ± 1.4	98 ± 1.4	510 ± 2
H2.5	0	0	0	0	252.8 ± 1.8	111 ± 1.3	535 ± 2
C-H2.5	0	0	0	0	249.1 ± 1.0	117 ± 1.2	537 ± 1
A-H2.5	0	0	0	0	250.5 ± 1.6	120 ± 1.4	536 ± 3
H5	0	0	0	0	249.7± 1.0	115 ± 1.1	539 ± 6
C-H5	0	0	0	0	248.8 ± 1.3	118 ± 1.4	538 ± 3
A-H5	0	0	0	0	249.6 ± 1.4	122 ± 1.5	537 ± 5
Ag	13	14	13	0	253.3 ± 1.4	105 ± 1.2	520 ± 3
H2.5-Ag	16	17	16	0	252.4 ± 1.2	114 ± 1.1	536 ± 4
C-H2.5-Ag	15	16	14	0	248.9 ± 1.1	119 ± 1.4	538 ± 5
A-H2.5-Ag	18	20	18	0	250.3 ± 1.9	122 ± 1.1	537 ± 4
H5-Ag	15	15	14	0	249.3 ± 1.1	117 ± 1.3	540 ± 3
C-H5-Ag	14	16	13	0	248.5 ± 1.5	120 ± 1.5	539 ± 2
A-H5-Ag	18	19	18	0	249.2 ± 1.1	124 ± 1.4	539 ± 5
